# Supplementary material for: Identifying the needs of people with long COVID: a qualitative study in the UK
Source: BMJ Open. 2024 Jun 19;14(6):e082728. doi: 10.1136/bmjopen-2023-082728 (PMC11191788; doi:10.1136/bmjopen-2023-082728)
Supplement: Supplementary data [file bmjopen-2023-082728supp001.pdf]

Supplementary Information

Guidance Questions followed by Facilitators

What symptoms post Covid are most distressing for you?

What can you not do now that you could do before Long Covid?

How has Long Covid affected your quality of life?

What can clinical services provide to help with your everyday functioning?

What services can society provide to help with your symptoms?

What self-help strategies have worked for you?

What research would most help pwLC get back to normal functioning?
